# Dab2ip Regulates Neuronal Migration and Neurite Outgrowth in the Developing Neocortex

**DOI:** 10.1371/journal.pone.0046592

**Published:** 2012-10-04

**Authors:** Gum Hwa Lee, Sun Hong Kim, Ramin Homayouni, Gabriella D'Arcangelo

**Affiliations:** 1 Department of Cell Biology and Neuroscience, Rutgers, The State University of New Jersey, Piscataway, New Jersey, United States of America; 2 Graduate Program in Molecular Biosciences, Rutgers, The State University of New Jersey, Piscataway, New Jersey, United States of America; 3 Department of Psychiatry and Behavioral Sciences, Johns Hopkins University, Baltimore, Maryland, United States of America; 4 Department of Biological Sciences, University of Memphis, Memphis, Tennessee, United States of America; Duke University Medical Center, United States of America

## Abstract

Dab2ip (DOC-2/DAB2 interacting protein) is a member of the Ras GTPase-activating protein (GAP) family that has been previously shown to function as a tumor suppressor in several systems. Dab2ip is also highly expressed in the brain where it interacts with Dab1, a key mediator of the Reelin pathway that controls several aspects of brain development and function. We found that Dab2ip is highly expressed in the developing cerebral cortex, but that mutations in the Reelin signaling pathway do not affect its expression. To determine whether Dab2ip plays a role in brain development, we knocked down or over expressed it in neuronal progenitor cells of the embryonic mouse neocortex using *in utero* electroporation. Dab2ip down-regulation severely disrupts neuronal migration, affecting preferentially late-born principal cortical neurons. Dab2ip overexpression also leads to migration defects. Structure-function experiments *in vivo* further show that both PH and GRD domains of Dab2ip are important for neuronal migration. A detailed analysis of transfected neurons reveals that Dab2ip down- or up-regulation disrupts the transition from a multipolar to a bipolar neuronal morphology in the intermediate zone. Knock down of Dab2ip in neurons *ex-vivo* indicates that this protein is necessary for proper neurite development and for the expression of several major neuronal microtubule associated proteins (MAPs), which are important for neurite growth and stabilization. Thus, our study identifies, for the first time, a critical role for Dab2ip in mammalian cortical development and begins to reveal molecular mechanisms that underlie this function.

## Introduction

During the early development of the mammalian cerebral cortex, principal neurons born in ventricular or subventricular zone migrate radially toward the pial surface and become positioned in defined cellular layers [1]. At early stages of corticogenesis, the majority of these neurons migrate by somal translocation, using their long leading processes contacting the pia to move efficiently into the upper cortical plate [2]. As the cortex develops further, the migration of later-born principal neurons becomes more complex. Many postmitotic neurons move up from the ventricular zone and acquire a multipolar morphology in the subventricular and intermediate zone [3], [4]. Here they pause until they acquire a bipolar morphology that enables them to migrate by glial-guided locomotion into the developing cortical plate. This mode of migration requires a leading process that wraps around a radial glia fiber, this latter serving a guidance scaffold [3], [4]. When the leading process of migrating neurons approaches the marginal zone, late-born neurons can also use somal translocation to become properly positioned in the superficial aspect of the developing cortex [2].

The molecular mechanisms that control the complex steps in neuronal migration have only partially been elucidated. Genetic studies in mouse models demonstrated that the extracellular protein Reelin (Reln) is required for the formation of cellular layers of the neocortex by regulating mostly somal translocation and neuronal orientation during radial migration [5]–[8]. The signal transduction machinery that mediates this activity of Reelin includes the ApoER2 and VLDLR receptors, and the adapter protein Dab1 [9]–[11]. Other molecules have been shown to regulate different steps of radial migration. For example, the microtubule-associated protein doublecortin (Dcx) and the lissencephaly protein Lis1 are required predominantly for glial-guided locomotion, by affecting the transition from multipolar to bipolar morphology [12]–[14]. In humans, mutations of genes including *RELN*, *VLDLR*, *LIS1*, and *DCX* all cause severe migration disorders and co-morbidities such as intellectual disability and epilepsy [15]–[19]. Despite significant recent advances in the field of brain development, our understanding of the mechanism underlying the pathophysiology of these disorders remains limited.

Dab2ip (DOC-2/DAB2 interacting protein, ASK1 interacting protein) is a member of the Ras GTPase-activating protein (GAP) family, which was initially identified for its growth inhibitory activity in prostate cancer cells [20], [21]. Dab2ip has been studied mainly as a tumor suppressor protein that controls cell proliferation, apoptosis, and cell survival through inhibition of Ras-Erk pathway, activation of Ask1-Jnk pathway, and inhibition of PI3K-Akt pathway respectively in several types of cancer [20], [22], [23]. Dab2ip is also abundantly expressed in the developing and adult brain, where it has been shown to interact with the Reelin transducing protein Dab1 [24]–[26]. However, its function in the brain has not been yet explored. Here we investigated the expression and the function of Dab2ip in the embryonic cerebral cortex using *in vivo* and *ex vivo* knock down and overexpression approaches. We show that Dab2ip is important for the migration of late-born principal neurons and for neurite development, affecting the transition to a bipolar morphology in the intermediate zone. The downregulation of Dab2ip was accompanied by a marked reduction in the expression of several microtubule-associated proteins (MAPs), thus providing a likely mechanism by which Dab2ip exerts its function in neuronal migration and maturation.

## Materials and Methods

### Animals handling and in utero electroporation (IUE)

All animals used in this study were handled in accordance with a protocol approved by the Association for Assessment and Accreditation of Laboratory Animal Care committee at Rutgers, the State University of New Jersey. Wild type mice (ICR mice, Taconic Farms) were used for *in utero* electroporation (IUE) experiments as described previously [27]. Approximately 2 µl of a plasmid mix (3 µg) containing Fast Green dye (Sigma) was injected into the lateral ventricle of embryonic day (E) 12.5 and 14.5 embryos, and electroporated using the ElectroSquarePorator ECM 830 (BTX) set at five 50 ms pulses of 40 V with 950 ms intervals. Embryos were allowed to develop *in utero* for 2–4 days after electroporation. Brains were then dissected, fixed overnight in 4% paraformaldehyde (PFA) at 4°C and then placed in 30% sucrose/1× PBS mix for cryoprotection. Brains were frozen in OCT (Tissue-Tek) and sectioned coronally at 30 µm for confocal analysis. All experiments were conducted at least in triplicates. Mutant mouse colonies were: *Reeler* mice (B6C3Fe-*ala-Relnrl*/+) (Jackson Laboratories, Bar Harbor, ME, USA), *Dab1* knock-out mice (a gift of J. A. Cooper, Fred Hutchinson Cancer Research Center, Seattle, WA), and conditional *Pten* knockout mice (NEX-*Pten*) [28].

### Expression constructs

Three different shRNA constructs targeting the Dab2ip coding sequence were generated using the mU6pro vector (a gift from Dr. David Turner, University of Michigan). The hairpin shRNA sequences are: 5′-GAGCACTTTGAGTTCCATAAC-3′, shDab2ip 1; 5′-GACCTCTCTGGTCTGATAGAT-3′, shDab2ip 2; 5′-GATATCAGTGAACGGCTCATC-3′, shDab2ip 3. As a non-targeting shRNA control, we used pGE-2-hrGFP II (Stratagene). pEGFP and pmCherry expression plasmids in which fluorescent proteins are encoded from the CAG promoter were described previously [27], [29]. Full-length Dab2ip cDNA (L isoform, GenBank accession number Q473307) was cloned by RT-PCR from mouse (C57B/6 and 129SvEv mixed genetic background) brain cDNA using BioXact polymerase (Biolase, UK) and primers with nested restriction enzyme sites (EcoRI forward primer: 5′-TAGAATTCGCCACCATGGAGCCCGACTCCCTCCTGGAC and NotI reverse primer: 5′-TAGCGGCCGCCTAATGCATACTCTCTTTCAGCTGTGT). The resulting PCR fragment was cloned into pcDNA3 mammalian expression vector and fully sequenced by primer walking strategy. The pcDNA3-Dab2ip-L plasmid was used as a template for generation of the full-length or truncated forms of Dab2ip into pCMV-Tag3B vector (Stratagene, La Jolla, CA, USA) and then the construct were moved to pCAG –IRES-EGFP vector (a gift from Mikio Hoshino, Kyoto University, Kyoto, Japan) for *in vivo* expression of Dab2ip constructs: pCAG-Myc-Dab2IP-L (aa 1–1132), pCAG-Myc-Dab2IP-LΔPH (aa 171–1132), pCAG-Myc-Dab2IP-LΔGRD (1–307/567–1132) was used. To generate point mutations Dab2ip GRD*, Dab2ip-L-R385L; Dab2ip NP/AA; Dab2ip-L m, a transcript resistant to Dab2ip shRNA degradation and a Dab2ip-LΔGRD construct, site-directed mutagenesis was performed using the QuickChange® XL kit (Stratagene).

### HEK 293T, COS-7, and primary cortical neuron culture and transfection

HEK 293T, COS-7 cells were grown and maintained in DMEM (high glucose; Invitrogen) with 10% fetal bovine serum (Invitrogen) at 37°C in 5% CO_2_. Expression and knock down constructs were delivered to these cells using Fugene 6 (Roche) according to the manufacturer's protocol. For primary cortical neuron cultures, cerebral cortices were dissected from E15.5 mouse ICR embryos. Neurons were dissociated using a Papain Dissociation Kit (BioWorthington, cat# LK003150). The cortical neuron cultures were maintained for 2–8 days *in vitro* (DIV) in Neurobasal medium supplemented with 0.5 mM l-glutamine, 2% B-27 supplement, 100 U/ml penicillin and 100 µg/ml streptomycin (Invitrogen), replacing half the medium after the first 3 days. To test knock down efficiency by Western blot analysis, approximately 5×10^6^ dissociated cells were transfected with shRNA constructs using an Amaxa nucleofector kit, and plated in a 6-well plate coated with poly-l-lysine. For Dab2ip protein localization experiments, dissociated cortical neurons were transfected by the calcium phosphate method at 5 DIV. For immunofluorescence analysis of neurons transfected by IUE at E14.5, cerebral cortices were dissected from E16.5 embryos, and the neurons were dissociated and cultured for 2 DIV. Approximately 0.2×10^6^ cells were placed on a glass coverslip in a 24-well plate coated with poly-l-lysine.

### Fluorescence analysis

Auto- and immunofluorescence analysis of brain tissue and dissociated cells was performed essentially as previously described [27]. Tissue sections were imaged by confocal microscopy using a Yokogawa CSU-10 spinning disk attached to an inverted fluorescence microscope (Olympus IX50). The dissociated cortical neurons transfected *in vivo* by IUE as described above were fixed in 4% PFA after 2–5 DIV. For Dab2ip immunofluorescence, E16.5 mouse brains were isolated, frozen on dry ice in OCT, and sectioned at 25 µm. Primary antibodies were: rabbit anti-Dab2ip (Abcam); rabbit anti-EGFP (Abcam); rabbit anti-Tbr1 (Millipore); rabbit anti-Map2 (Millipore); goat anti-Map1b (Santa Cruz); goat anti-Tau (Santa Cruz); mouse anti-Tuj1 (Covance); rabbit anti-Myc (Sigma); mouse anti-Satb2 (Abcam); mouse anti-Nestin (Millipore). Secondary antibodies were conjugated to AlexaFluor 488, AlexaFluor 647, and Cy5 (Invitrogen).

### Western blot analysis

Primary cortical neurons, HEK 293 T cells, and the cerebral cortex of wild type or mutant mice were lysed in RIPA buffer (50 mM Tris pH 7.4, 1% NP40, 0.25% deoxycholate, 150 mM NaCl, 1 mM EGTA) and cleared by centrifugation at 3,000× *g* for 3 min at 4°C. 5 µg of HEK 293 T lysate and 15 µg of cortical neuron lysate were loaded onto 8% or 12% SDS-PAGE gels, run at 130 V for 2 h, and transferred to 0.22 µm nitrocellulose membranes. The membranes were blocked with 3% milk in 1× TBS-T (Tris-buffered saline with 0.1% Tween 20) for 1 h at room temperature, followed by incubation with primary antibodies overnight at 4°C, and incubation with secondary antibodies for 1 h at room temperature. After several washes with TBS-T, membranes were developed with ECL-Plus Western Blotting Detection System (GE Healthcare) and exposed to autoradiographic film (Denville). Primary antibodies were: rabbit anti-Dab2ip (Abcam), mouse monoclonal anti-Dab1 (a gift from Dr. André M Goffinet, Université Catholique de Louvain, Belgium), rabbit phospho-Akt (Serine 473) (Cell Signaling), and mouse anti-actin (Millipore), mouse anti-Myc (Santa Cruz). Secondary antibodies were HRP-conjugated (Sigma).

### Statistical analysis

Data in the plots are shown as the mean +/− s.e.m., and analyzed by Student's *t* test or one-sample *t*-test as indicated in the figure legends. To count GFP+ cells in brain sections after IUE, median sections were chosen in a series of GFP+ sections, and the results were averaged from multiple sections per embryos. Statistical significance was determined when *p*<0.05.

## Results

### Dab2ip expression in the developing neocortex of wild type and mutant mice

**Figure 1:**
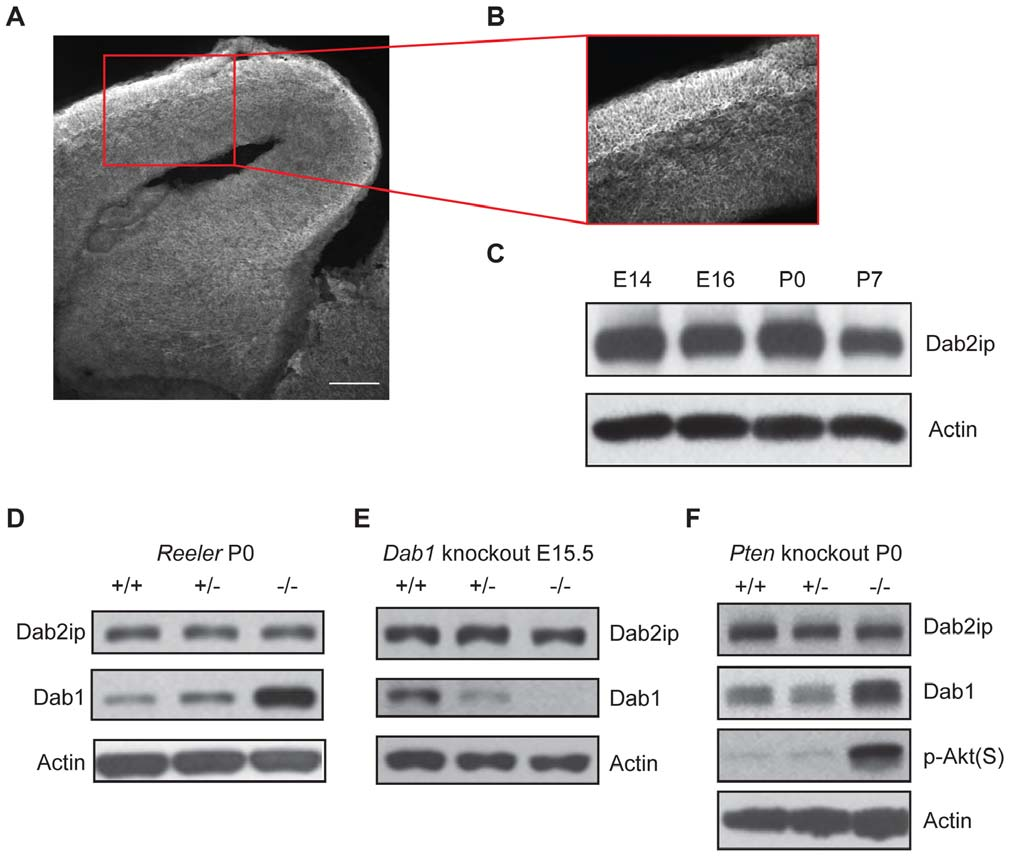
Dab2ip expression in the developing neocortex of wild type and mutant mice. (A, B) Brain sections obtained from E16.5 mice were immunostained with anti-Dab2ip antibodies. The image in (B) is a magnification of the inset shown in (A). (C) Western blot analysis was performed using neocortex from ICR mice at the indicated embryonic or postnatal ages. The same blot was first probed with Dab2ip antibodies, and then was reprobed with antibodies against actin as an internal loading control. Dab2ip was expressed at all ages throughout the neocortex. The neocortex of newborn wild type (+/+), heterozygous (+/−) and homozygous (−/−) *reeler* littermates (D), embryonic *Dab1* knockout mice (E), and newborn *Pten* conditional knockout mice (F), was analyzed. Western blots were sequentially probed with Dab2ip, Dab1, phospho- Akt Serine 473 (p-Akt(S)) and actin antibodies. Levels of Dab2ip, unlike Dab1 and pAkt(S), did not differ among genotypes. Scale bars: 100 µm (A), 50 µm (B).

To investigate whether Dab2ip is involved in brain development, we began by analyzing its expression in the developing mouse neocortex by immunofluorescence and Western blot analysis. Confocal analysis of brain sections obtained from embryonic day (E) 16.5 mice revealed that Dab2ip protein is expressed throughout the developing neocortex, at particularly higher levels in the cortical plate, where principal cortical neurons migrate and mature (Fig. 1A and B). This expression pattern is consistent with previous studies that described the expression of Dab2ip mRNA in the developing mouse neocortex [26], [30]. Western blot analysis further demonstrated that Dab2ip protein is abundantly expressed in this region from E14.5 to at least postnatal day (P) 7 (Fig. 1C). Even though multiple isoforms of Dab2ip of different molecular weights have been previously described [20], [24], [25], in our experiments Dab2ip appeared as a single band of approximately 110 kDa (Fig. 1C), suggesting that this protein isoform is predominant in the embryonic and early postnatal neocortex. Since Dab2ip has been shown to interact with Dab1, a critical adaptor molecule in the Reelin signaling pathway that controls neuronal migration and maturation, we investigated Dab2ip expression in several mutant mice carrying mutations in this pathway. Reelin induces the phosphorylation of Dab1 on tyrosine residues, and event that is coupled to the ubiquitination and degradation of Dab1 [31]–[34]. Thus, Dab1 protein levels are increased in homozygous *reeler* mice lacking Reelin [31] (Fig. 1D). We found that Dab2ip levels, however, did not change in the brain of neither *reeler* mice (Fig. 1D) nor Dab1 knock out mice (Fig. 1E). Because Reelin is known to stimulate the activity of the phosphatidylinositol 3-kinase (PI3K) pathway through a mechanism that requires Dab1 [35], and Dab1 levels are increased in mouse mutants that lack the upstream phosphatase Pten [28], we also examined Dab2ip expression in the neocortex of Pten knockout mice. We found that, although levels of Dab1 and phosphoAkt were noticeably increased in homozygous Pten mutants, levels of Dab2ip were not affected (Fig. 1F). Together, these data indicate that Dab2ip protein expression and stability are not modulated by the Reelin pathway.

### Generation and validation of Dab2ip shRNA constructs

**Figure 2:**
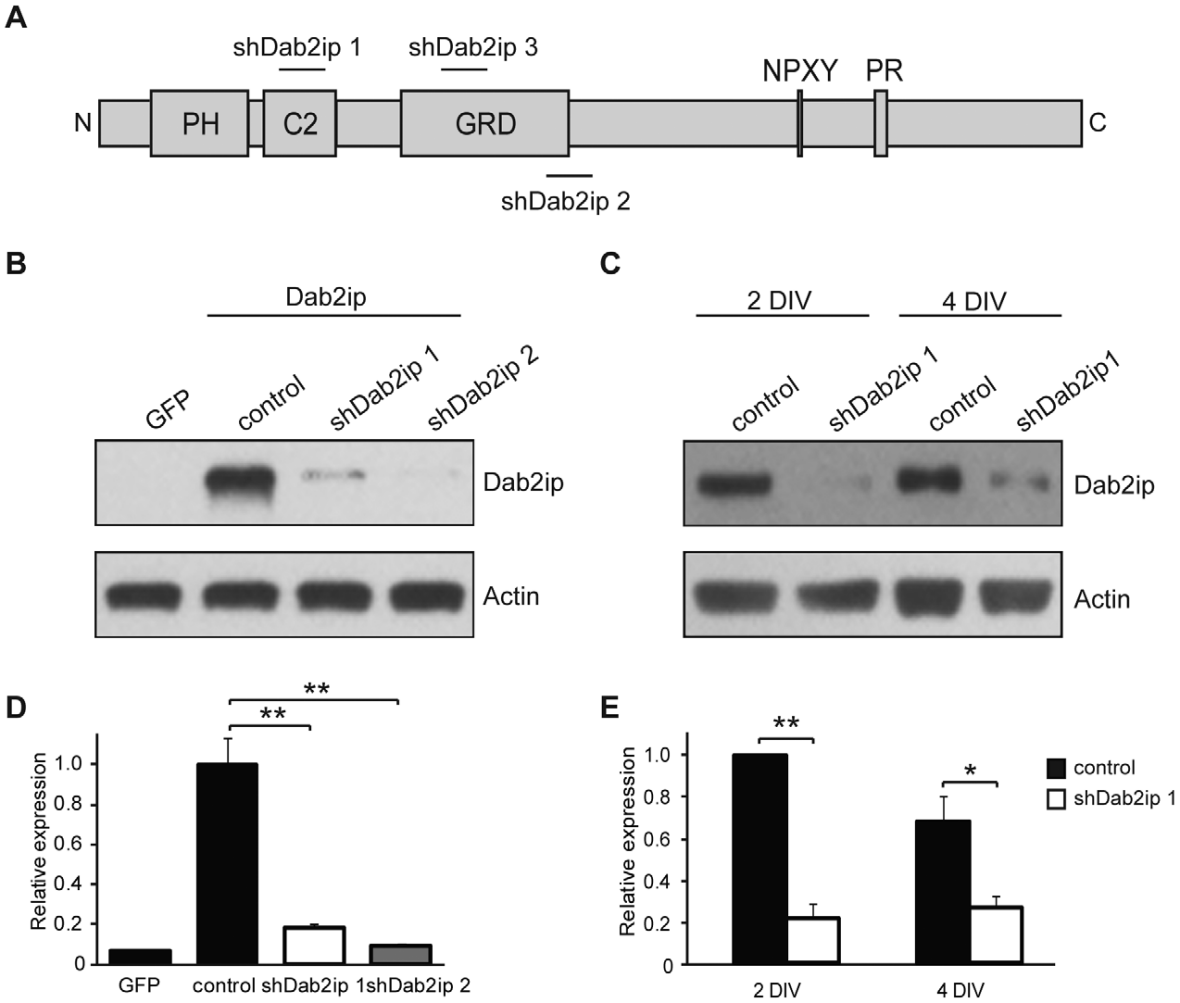
Knock down of Dab2ip by shRNA in transfected and primary cells. (A) Diagram of Dab2ip, indicating protein functional domains and the targeted regions of Dab2ip shRNAs shDab2ip 1, shDab2ip 2, and shDab2ip 3. PH: Pleckstrin homology, C2: phospholipid/Ca2+ binding motif, GRD: GAP-related domain, NPxY: internalization and Dab1/Dab2 binding motif, PR: proline-rich domain. (B, D) Knock down of exogenous Dab2ip by shDab2ip 1 and shDab2ip 2 in transfected HEK 293T cells. Cells were transfected with a GFP plasmid or co-transfected with a plasmid encoding Dab2ip (isoform L) and a non-targeting shRNA (control) or Dab2ip shRNAs 1 or 2. Both specific shRNAs strongly reduced Dab2ip expression. The plot in (D) shows the results from triplicate experiments. Dab2ip levels were normalized to actin and expressed relative to the control shRNA. Statistical significance was determined by the Student's *t*-test. (C, E) Knock down of endogenous Dab2ip by shDab2ip 1 in primary cortical neurons. Neurons were transfected with non-targeting shRNA (control) or shDab2ip 1, and analyzed at 2 DIV and 4 DIV. The plot in (E) shows the results from triplicate experiments. The data was analyzed as above, except that statistical significance was determined by a one-tailed *t*-test. *, *p*<0.05; **, *p*<0.01.

To explore the function of Dab2ip in cortical development, we first isolated complementary RNA from the embryonic mouse cerebral cortex. Partial sequencing of the 5′ end of cloned PCR products indicated that embryonic cortical Dab2ip transcripts contained, in addition to the GAP-related domain (GRD) and the phospholipid/Ca^++^ binding motif (C2) found in all known isoforms of Dab2ip, a pleckstrin homology (PH) domain that is present in most, but not all, cloned isoforms. The cortical isoform lacked additional N terminal regions present in the long Dab2ip (isoform L) (GenBank DQ473307). To knock down Dab2ip expression during brain development, we generated short hairpin RNAs (shRNAs). The target regions of these shRNAs, referred as shDab2ip 1, shDab2ip 2, and shDab2ip 3, are shown relative to the Dab2ip protein diagram (Fig. 2A). To test the efficacy of these constructs, HEK 293T cells were transfected with a plasmid encoding mouse Dab2ip (isoform L), or GFP as a negative control. Dab2ip-transfected cells were co-transfected with a non-targeting shRNA (control), shDab2ip 1, or shDab2ip 2. The data, quantified from triplicate experiments, indicate that both, shDab2ip 1 and shDab2ip 2 dramatically reduce exogenous Dab2ip expression (Fig. 2B and D). To examine the effectiveness of these constructs on endogenous Dab2ip expression, primary cortical neurons were transfected with plasmids encoding control shRNA or shDab2ip 1 using the Amaxa nucleofector system. Levels of Dab2ip expression were analyzed after 2 or 4 days *in vitro* (DIV). Quantification of the data indicates that Dab2ip protein expression was dramatically reduced by shDab2ip 1 compared to control shRNA at both time points analyzed, and that the knock down efficiency reached approximately 78% at 2 DIV (Fig. 2C and E). This represents a dramatic down-regulation of Dab2ip since in parallel experiments the transfection efficiency of cortical neurons using a control GFP plasmid was estimated to be approximately 80% (not shown). Quantitative RT-PCR analysis also confirmed that Dab2ip mRNA was dramatically reduced by shDab2ip 1 (data not shown).

### Dab2ip is required for cortical neuron migration

**Figure 3:**
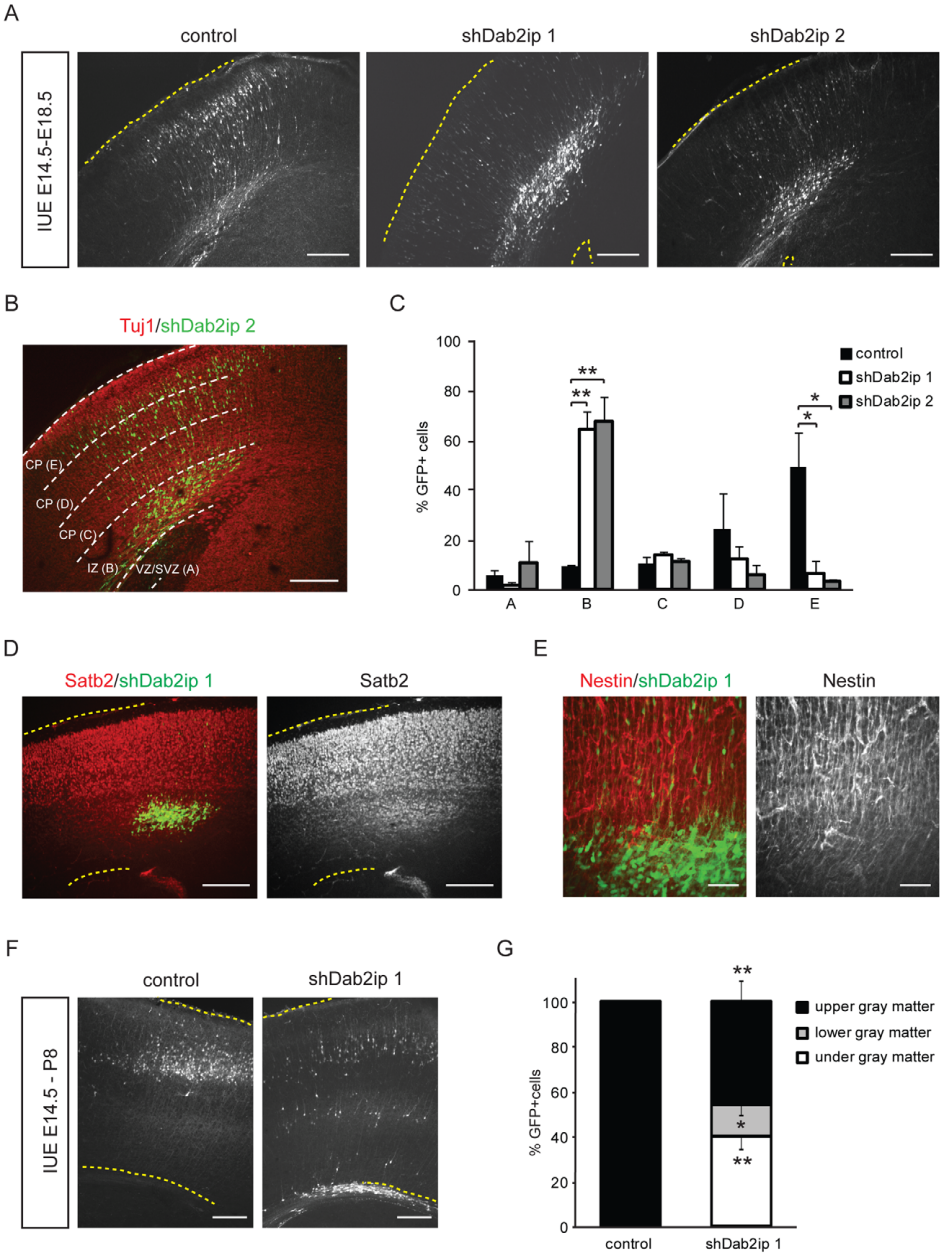
Dab2ip is required for cortical neuron migration. Control shRNA or shDab2ip 1 and 2 were trasnfected into neural progenitor cells of the mouse embryonic neocortex at E14.5 by IUE. The migrating cortical neurons were visualized by co-transfection with a GFP-expressing plasmid. (A) Representative confocal images of the lateral neocortex 4 days after IUE with the indicated shRNA constructs show the distribution of GFP+ cells. The dotted lines indicate the pial surface. Dab2ip knock down neurons failed to migrate, whereas control neurons migrated efficiently toward the surface of the neocortex. (B) GFP autofluorescence (green) and Tuj1 immunofluorescence (red) were used to quantify neuronal migration. Images of the electroporated neocortex were divided into five equal-interval bins. The lowest bin A corresponds to the Tuj1− VZ/SVZ region; bin B corresponds to the IZ and contains many Tuj1+ cells; bins C, D, and E correspond to increasing superficial regions of CP, containing heavily Tuj1+ cells. (C) The percentage of GFP+ neurons in each bin was calculated from multiple electroporated embryos. In the embryos transfected with shDab2ips, most neurons were confined in the IZ (control, 9.5%, *n* = 3; shDab2ip 1, 64.5%, *n* = 3; shDab2ip 2, 67.6%, *n* = 3), and failed to migrate into the CP (control, 49.5%; shDab2ip 1, 6.8%; shDab2ip 2, 3.4%). (D) Sections of brains electroporated with shDab2ip 1 (green) at E14.5 were double-labeled with Satb2 antibodies (red). Arrested knock down neurons in the IZ expressed the Satb2 upper layer marker. (E) Sections adjacent to those imaged in D were double-labeled with Nestin antibodies (red) to label the radial glia scaffold surrounding Dab2ip knock down neurons (green). (F) Representative confocal images of the lateral neocortex of mice transfected at E14.5 and analyzed at postnatal day 8. The neocortical grey matter was divided in two equal bins to quantify the distribution of transfected neurons in upper or lower regions. Many GFP+ neurons transfected with shDab2ip 1, unlike those transfected with control shRNA, remained localized underneath cellular layers (control, 0%, *n* = 3; shDab2ip 1, 40.8%, *n* = 3) or in deep layers. CP = cortical plate; IZ = intermediate zone; VZ = ventricular zone; SVZ = subventricular zone. Scale bars: 200 µm (A, B, D, and F), 50 µm (E) *, *p*<0.05; **, *p*<0.01.

To investigate the function of Dab2ip in corticogenesis, we performed *in utero* electroporation (IUE) experiments to introduce the Dab2ip shRNAs or a control shRNA into neural progenitor cells of the embryonic mouse neocortex. Plasmids encoding shRNAs were co-transfected with a GFP expression construct to facilitate detection of the transfected cells. In embryos electroporated with control shRNA at E14.5, the great majority of neurons generated from GFP+ progenitor cells entered the subventricular zone (SVZ) and the intermediate zone (IZ) 2 days after electroporation (Fig. S1, A and B), invaded the cortical plate (CP) 3 days (Fig. S1, C and D) and reached the upper CP 4 days (Fig. 3A) after electroporation. In embryos electroporated with shDab2ip 1 at E14.5, most GFP+ neurons also entered the SVZ and the IZ 2 days later (Fig. S1A), but many failed to invade the CP 3 days (Fig. S1B) and 4 days after electroporation (Fig. 3A). Many knock down GFP+ neurons appeared to be arrested below the CP, although a few were able to migrate fairly normally. Similar results were obtained using shDab2ip 2 (Fig. 3A) and shDab2ip 3 (not shown). In order to quantify our IUE results, we double-labeled E18.5 embryonic sections by immunofluorescence using antibodies against the early neuronal marker β-III tubulin (Tuj1), which highlights different regions of the developing cortex. Confocal images of the lateral neocortex were divided into five equal-interval bins, and the percentage of GFP^+^ cells distributed in each bin was determined from multiple comparable sections obtained from *n* = 3 embryos per construct. With this method, the lowest bin (A) roughly corresponds to the Tuj1-negative SVZ/VZ, the second lowest bin (B) corresponds to the IZ containing many Tuj1-labeled processes, and the other bins (C–E) correspond to increasingly superficial regions of the CP, which were heavily Tuj1-positive (Fig. 3B). We found that the majority of control neurons (84.6%) migrated into the CP in the E14.5–18.5 time frame, and approximately 50% of these neurons reached the most superficial CP bin E (Fig. 3C). On the other hand, neurons in which Dab2ip expression had been knocked down remained mostly confined to the IZ (shDab2ip 1: 64.5% in bin B; shDab2ip 2: 67.6%; versus control shRNA: 9.5%) (Fig. 3C). Some knock down neurons were able to reach the CP, but very few of these neurons were found in superficial areas (shDab2ip 1: 6.8% in bin E; shDab2ip 2: 3.4%; versus control shRNA: 49.5%). The difference between control and either, shDab2ip 1 or shDab2ip 2 was statistically significant in bins E and B (*p*<0.05 and *p*<0.01, respectively). The failure of E14.5 knock down neurons to reach the cortical plate could result from impaired migration, differentiation or depletion of the radial glia scaffold that is necessary for locomotion. To distinguish between these possibilities, we stained electroporated brain sections with antibodies against the upper layer marker Satb2 and the radial glial marker Nestin. The data indicate that many Dab2ip knock down neurons expressed Satb2, suggesting that they are able to acquire an upper layer specification despite their abnormal positioning (Fig. 3D). Nestin immunofluorescence further indicate that a radial glia scaffold is present in cortical areas that contains many Dab2ip knock down neurons (Fig. 3E). Together, these data suggest that an intrinsic migration defect is primarily responsible for the abnormal localization of knock down neurons. To determine whether Dab2ip knock down caused a permanent migration arrest or a transient delay in migration, we also electroporated embryos at E14.4 and analyzed brains at postnatal day 8. At this age, virtually 100% of control transfected neurons were appropriately localized to the upper region of the neocortex, approximately corresponding to cellular layers 2–4 (Fig. 3F and G). Instead, only approximately half of the neurons transfected with Dab2ip shRNA reached the proper destination in the upper neocortex, whereas many remained localized underneath cellular layers (40.4%) or in the lower grey matter (13.6%) (Fig. 3F and G). These data indicate that Dab2ip knock down in progenitor cells destined to give rise to upper layer cortical neurons causes a persistent radial migration defect.

**Figure 4:**
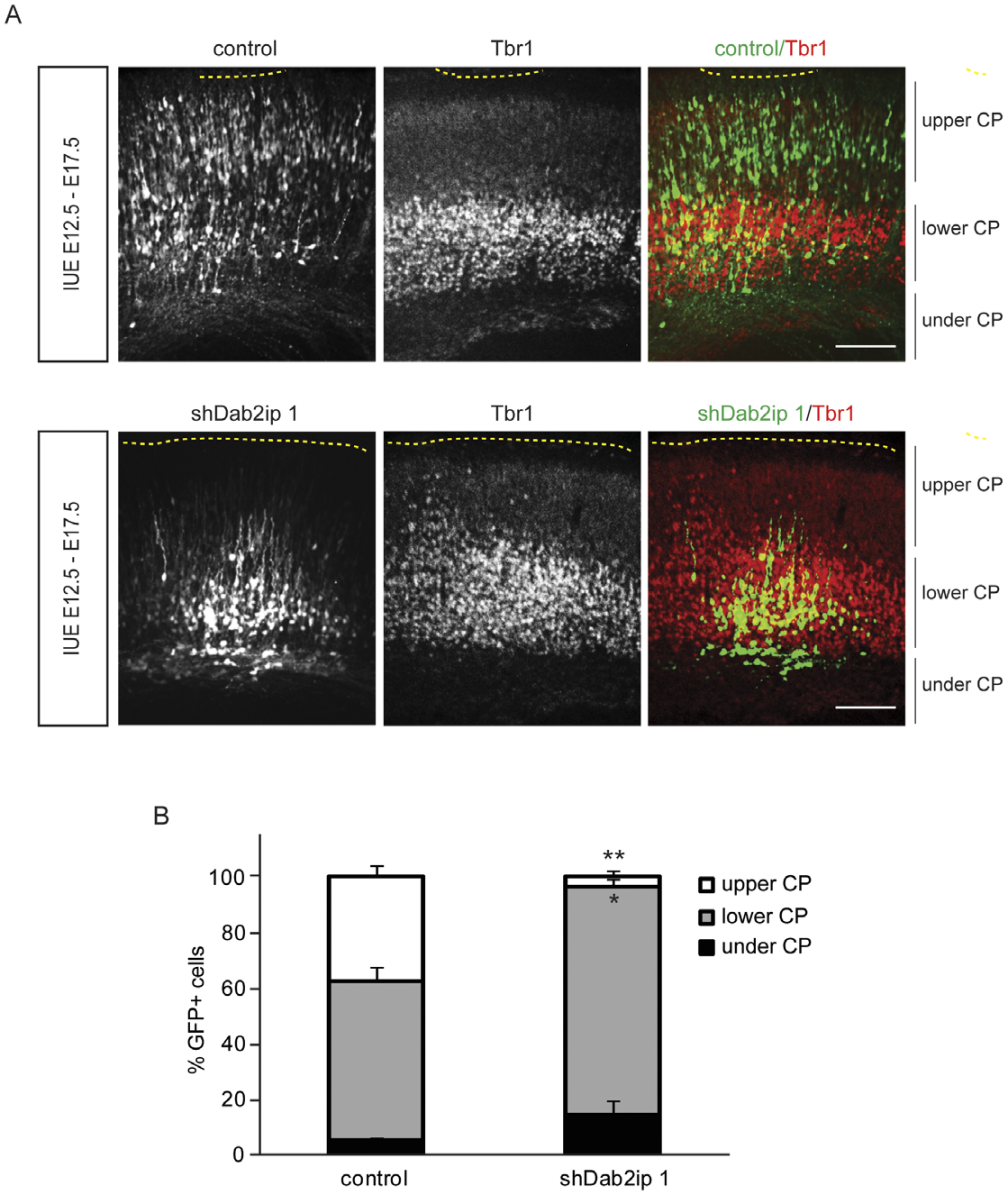
Dab2ip does not affect to the migration of early-born cortical neurons. Mouse embryos were electroporated at E12.5 with a GFP-expressing plasmid and control shRNA or shDab2ip 1. Sections of the caudal neocortex were analyzed at E17.5 by GFP autofluorescence (green) and immunofluorescence with Tbr1 antibodies (red). (A) Representative confocal images of the lateral neocortex show that Tbr1+ early-born knock down neurons localize appropriately to the lower CP, whereas Tbr1− late-born neurons fail to migrate to the upper CP. (B) Quantification of the data from multiple experiments. The cortical plate was divided in three bins based on the Tbr1 immunofluorescence signal. Upper CP = region above the Tbr1+ layer; lower CP = region containing most Tbr1+ neurons; under CP = region below the Tbr1+ layer. Scale bars: 100 µm *, *p*<0.05; **, *p*<0.01.

To determine whether Dab2ip also affects the migration of early-born neurons destined for deep cortical layers, we electroporated embryos at E12.5 with a GFP expression construct and either control or Dab2ip shRNA, and examined the distribution of GFP+ cells in the neocortex at E17.5. Electroporation at this age causes GFP expression in both, deep layer neurons born at E12–13 from the first round of progenitor cell division, as well as upper layer neurons born E14–17 from subsequent cell division events. To facilitate the identification of deep cortical layers we stained brain sections with Tbr1 antibodies and examined caudal regions of the neocortex where Tbr1+ cells are predominantly confined to deep layers. In embryos electroporated with control shRNA, GFP+ neurons that co-labeled with Tbr1 (early-born neurons) were appropriately located in deep layers, whereas Tbr1− neurons (late-born neurons) invaded the upper layers (Fig. 4A and B). In contrast, GFP+ neurons transfected with shDab2ip 1 were localized exclusively either in deep cortical layers (mostly Tbr1+ neurons) or underneath the CP (Tbr1− neurons) (Fig. 4A and B). These data strongly suggest that Dab2ip knock down preferentially affects the migration of upper cortical neurons.

### Dab2ip overexpression and structure-function analysis *in vivo*

**Figure 5:**
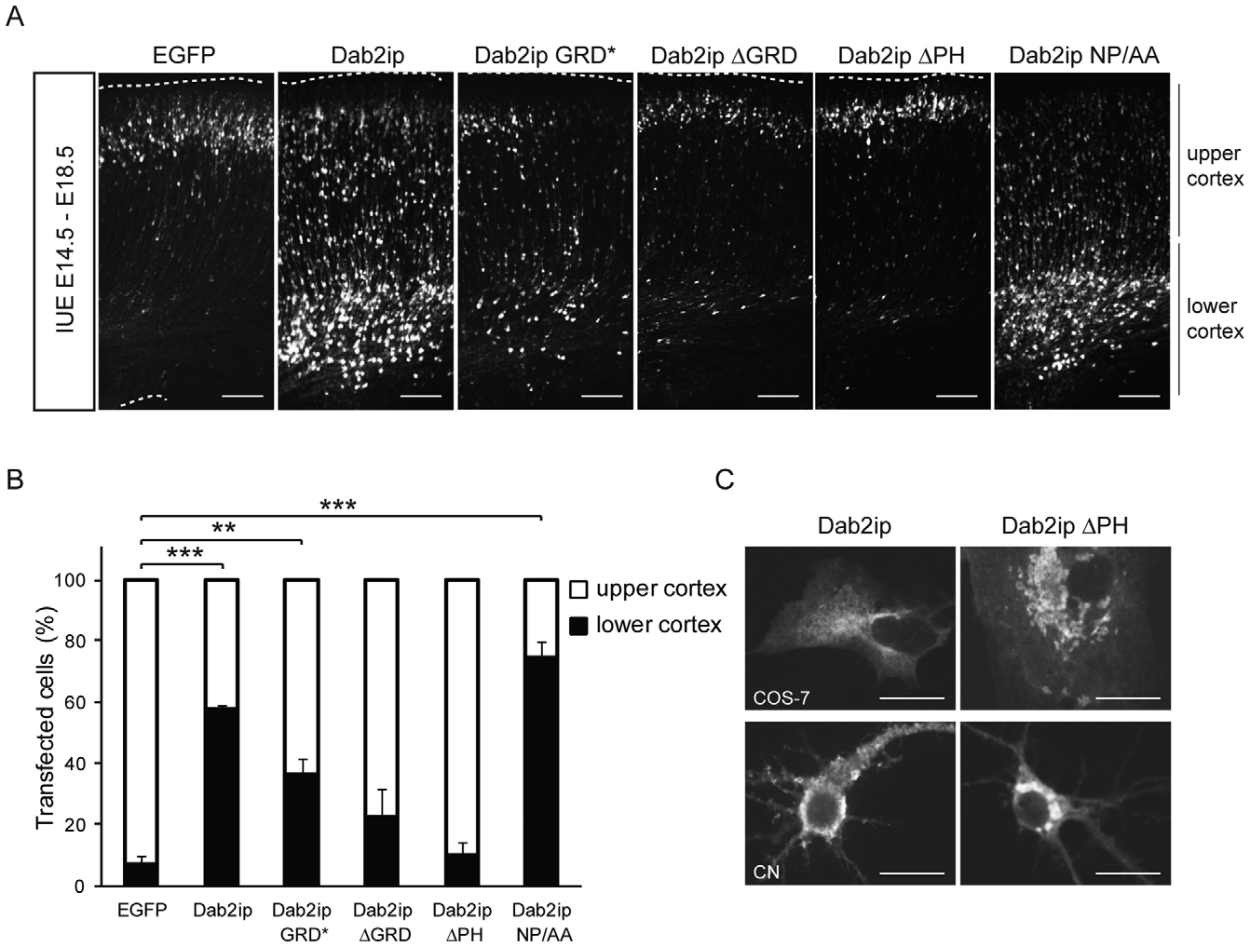
Dab2ip overexpression causes migration defects that require intact PH domain and GRD, but not the NPxY motif. (A) Mouse embryos were electroporated at E14.5 with the indicated Dab2ip expression constructs. Representative confocal images of GFP+ neurons in the developing cortex at E18.5 show that exogenous expression of intact Dab2ip or a construct in which the NPxY motif was mutated cause obvious migration defects. The phenotype was attenuated when constructs carrying a mutation or deletions in the PH domain or GRD were used. (B) Quantification of the data from multiple experiments. The neocortex was divided in two equal bins corresponding to the upper and lower cortex. (C) COS-7 cells and primary cortical neurons were transfected with Myc-tagged Dab2ip of Dab2ipΔPH constructs and processed by immunofluorescence of with anti-Myc antibody. COS-7 cells were analyzed 2 days after transfection and primary cortical neurons were analyzed 3 after transfection. Scale bars: 100 µm (A), 20 µm (C) **, *p*<0.01; ***, *p*<0.001.

To determine whether increased Dab2ip expression also affects neuronal migration in the neocortex, we electroporated a construct encoding full-length mouse Dab2ip (L isoform) and several deletion or mutation constructs of Dab2ip into the brain of mouse embryos. The constructs include: Dab2ip GRD*, a point mutation in the GRD that abolishes that GTPase activity; Dab2ip ΔGRD, a deletion of GRD; Dab2ip ΔPH, a deletion of the PH domain; Dab2ip NP/AA, a double point mutation of the NPxY motif that is predicted to mediate Dab1 interaction [20], [26]. Western blot analysis confirmed that mutant and wild type Dab2ip proteins were expressed at comparable levels (Fig. S2). Constructs were co-electroporated with a GFP expression construct at E14.5 and neuronal migration was analyzed 4 days later. At this age, the great majority of control GFP+ electroporated neurons were found in the upper CP (Fig. 5A and B). Instead, more than half of the neurons transfected with the intact Dab2ip construct were unable to reach the upper CP and many stalled around the SVZ/IZ boundary (Fig. 5A and B). This migration defect was completely abolished by the deletion of the PH domain, and was also partially reduced by the GRD deletion or mutation (Fig. 5A). On the other hand, mutation in the NPxY motif resulted in a migration defect that was similar or even more dramatic than that of intact Dab2ip (Fig. 5A), suggesting that it did not result from interference with Dab1 signaling. Since the PH domain is known to bind phospholipids [36], we examined the cellular localization of Dab2ip ΔPH compared to intact Dab2ip in COS-7 cells and primary cortical neurons. The data show that Dab2ip ΔPH was localized preferentially around the nucleus whereas intact Dab2ip was dispersed evenly throughout the cytoplasm and the plasma membrane in both cell types (Fig. 5C). These findings suggest that the cellular localization of Dab2ip, mediated by its PH domain, is crucial for the function of this protein in neuronal migration, and that the GTPase activity also plays an important role in this activity.

### Dab2ip levels affect neuronal morphology *in vivo*

**Figure 6:**
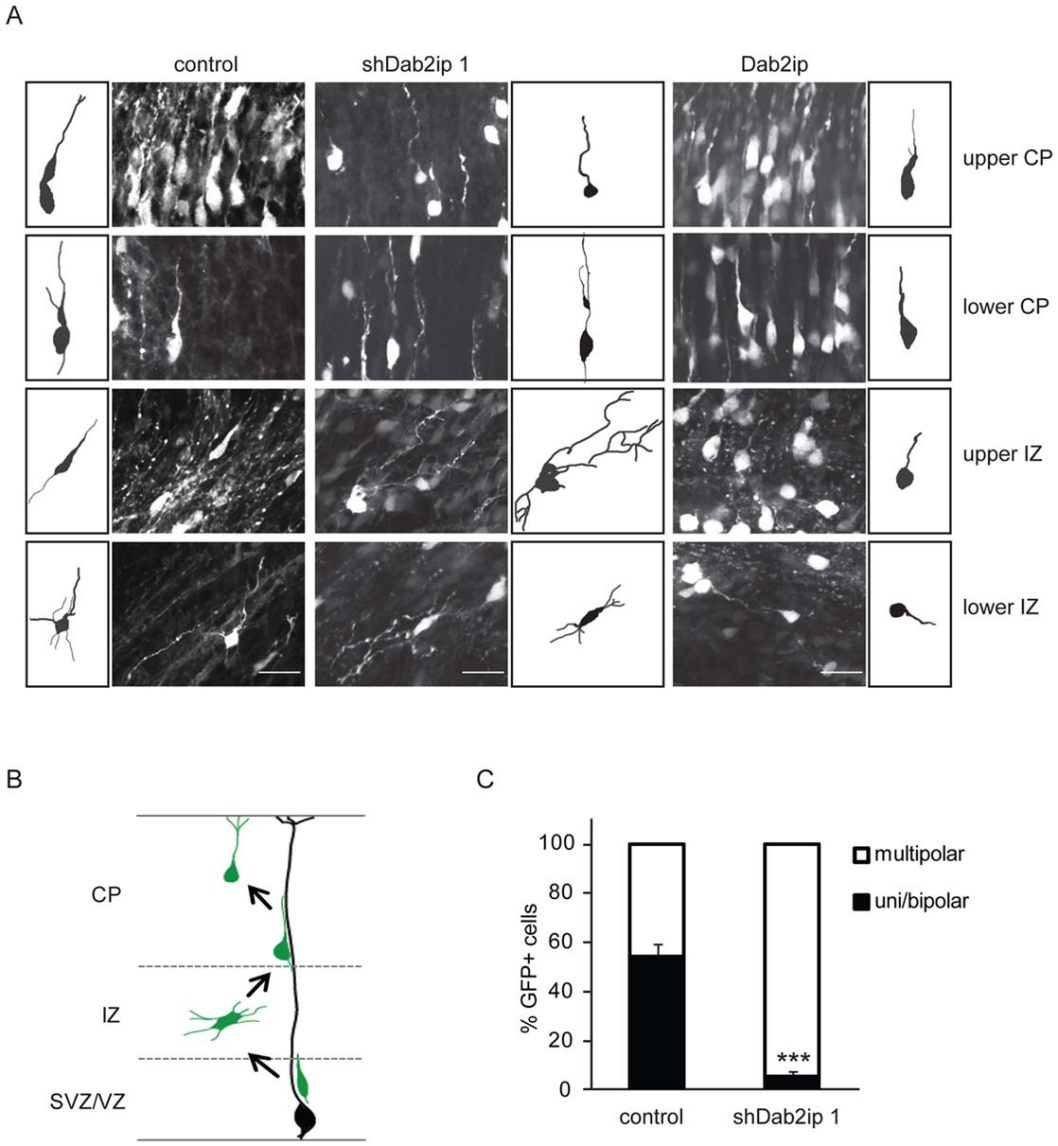
Dab2ip plays a role in the transition from multipolar to bipolar morphology during radial migration. (A) E14.5 embryos were electroporated *in utero* with control shRNA, shDab2ip 1 or a Dab2ip expression plasmid, and analyzed at E18.5. High magnification confocal images and tracing of individual, representative GFP+ neurons are shown in different regions of the developing neocortex, as indicated in each row. (B) Diagram of the morphological changes expected in each cortical region during the migration of late-born neurons. Neurons born in the VZ or SVZ move radially into the IZ where they acquire a multipolar morphology. They then convert to a bipolar morphology and undergo glial-guided locomotion to enter the CP. (C) Quantification of the percentage of multipolar neurons in the IZ. The data were obtained from confocal images of multiple sections as in (A). A significantly higher percentage of knock down neurons in the IZ exhibited a multipolar morphology compared to control. Scale bars: 25 µm ***, *p*<0.001.

The majority of principal cortical neurons destined for upper layers initially move radially from the VZ/SVZ into the IZ where they pause and acquire a multipolar morphology [3]. These cells then convert to a bipolar morphology and undergo glia-guided locomotion to enter the CP (Fig. 6B). To investigate the effect of Dab2ip up- or down-regulation on neuronal morphology in the developing neocortex, we electroporated Dab2ip expression or knock down plasmids constructs *in utero* at E14.5. Neurons destined for upper cortical layers were identified by GFP co-transfection, and analyzed by confocal microscopy 4 days after electroporation. At this time, most GFP+ control neurons had reached the upper CP. The few control neurons still located in the IZ displayed predominantly a bipolar morphology in the upper IZ and a multipolar morphology in the lower IZ (Fig. 6A). GFP+ Dab2ip knock down neurons were mostly confined in the IZ, where they exhibited a multipolar morphology in both, the upper as well as the lower IZ (Fig. 6A). The few knock down neurons that were able to reach the CP exhibited neuritic processes that were slightly coiled and thinner than control neurons (Fig. 6A). To quantify the morphological defect due to Dab2ip knock down, we repeated these experiments and calculated the percentage of multipolar and uni/bipolar neurons in the IZ 3 days after IUE with shDab2ip 1 or control shRNA. At this time point, approximately half of the GFP+ control neurons displayed uni/bipolar morphology in the upper IZ, whereas the other half exhibited a multipolar morphology and was mostly confined to the lower IZ (Fig. 6C). In contrast, the great majority of GFP+ knock down neurons in the IZ exhibited a multipolar morphology (94.5%, *n* = 3 embryos) (Fig. 6C). GFP+ neurons overexpressing Dab2ip also displayed an abnormal morphology, which was different than that produced by either control or knock down neurons. Overexpressing neurons exhibited a round soma, and often a single neuritic process (Fig. 6A). Multipolar neurons could not be identified even in the lower IZ. Together, these observations suggest that normal levels of Dab2ip promote the transition from multipolar to bipolar morphology, which is necessary for efficient glial-guided migration of cortical neurons destined to upper cortical layers.

### Dab2ip knock down disrupts neurite development in cultured neurons

**Figure 7:**
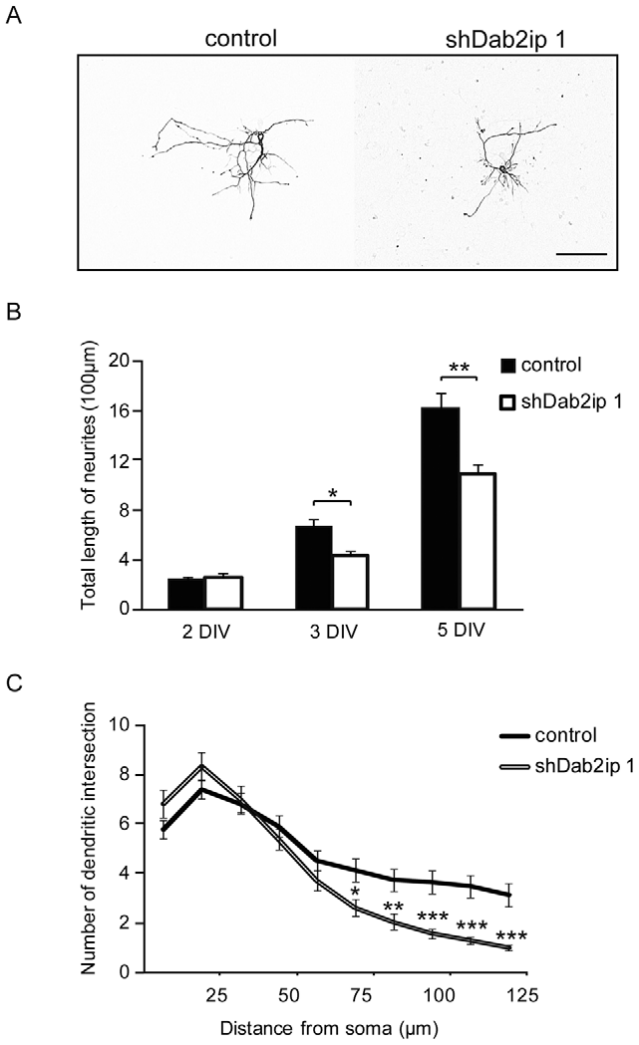
Dab2ip knock down disrupts neurite outgrowth in cultured neurons. Cortical neurons were isolated 2 days after *in utero* electroporation and cultured up to 5 days *in vitro*. (A) Representative images of transfected neurons at 5 DIV using ‘cost’ application in ImageJ. (B) The total length of neurites of Dab2ip knock down neurons and control neurons at 2 DIV, 3 DIV, and 5 DIV was measured using the NeuronJ application in ImageJ. The length of knock down neurites was significantly reduced than control at 3 and 5 DIV. (C) Sholl analysis of transfected neurons at 5 DIV indicates that knock down neurites were significantly shorter than control. Scale bars: 100 µm *, *p*<0.05; **, *p*<0.01; ***, *p*<0.001.

To further examine the role of Dab2ip in neurite development, we isolated cortical neurons 2 days after *in utero* electroporation (IUE E14.5–E16.5) with either shDab2ip 1 or control shRNA, and co-cultured them for up to 5 DIV. To distinguish knock down from control neurons, embryos were co-transfected either with plasmids encoding shDab2ip 1 and GFP alone, or with plasmids encoding control shRNA and GFP plus mCherry. After 2, 3, or 5 DIV, we imaged knock down (GFP+) and control (GFP+ and mCherry+) neurons by confocal microscopy, and analyzed their morphology in detail. At 2 DIV, the complexity and the total length of neurites did not differ between knock down and control neurons (Fig. 7B). However, by 3 DIV, Dab2ip knock down neurons showed a significant decrease in the total length of neurites compared to control (Fig. 7B). This difference became more pronounced at 5 DIV, at which time the processes of Dab2ip knock down neurons also appeared thinner than control (Fig. 7A and B). To further examine neurite complexity, we conducted Sholl analysis of transfected neurons at 5 DIV. This analysis indicated that the neurites of Dab2ip knock down neurons were slightly more numerous, but significantly shorter than control (Fig. 7C). These *in vitro* results are consistent with our *in vivo* observations and confirm the role of Dab2ip in neurite elongation.

### Dab2ip is required for the expression of microtubule–associated proteins

**Figure 8:**
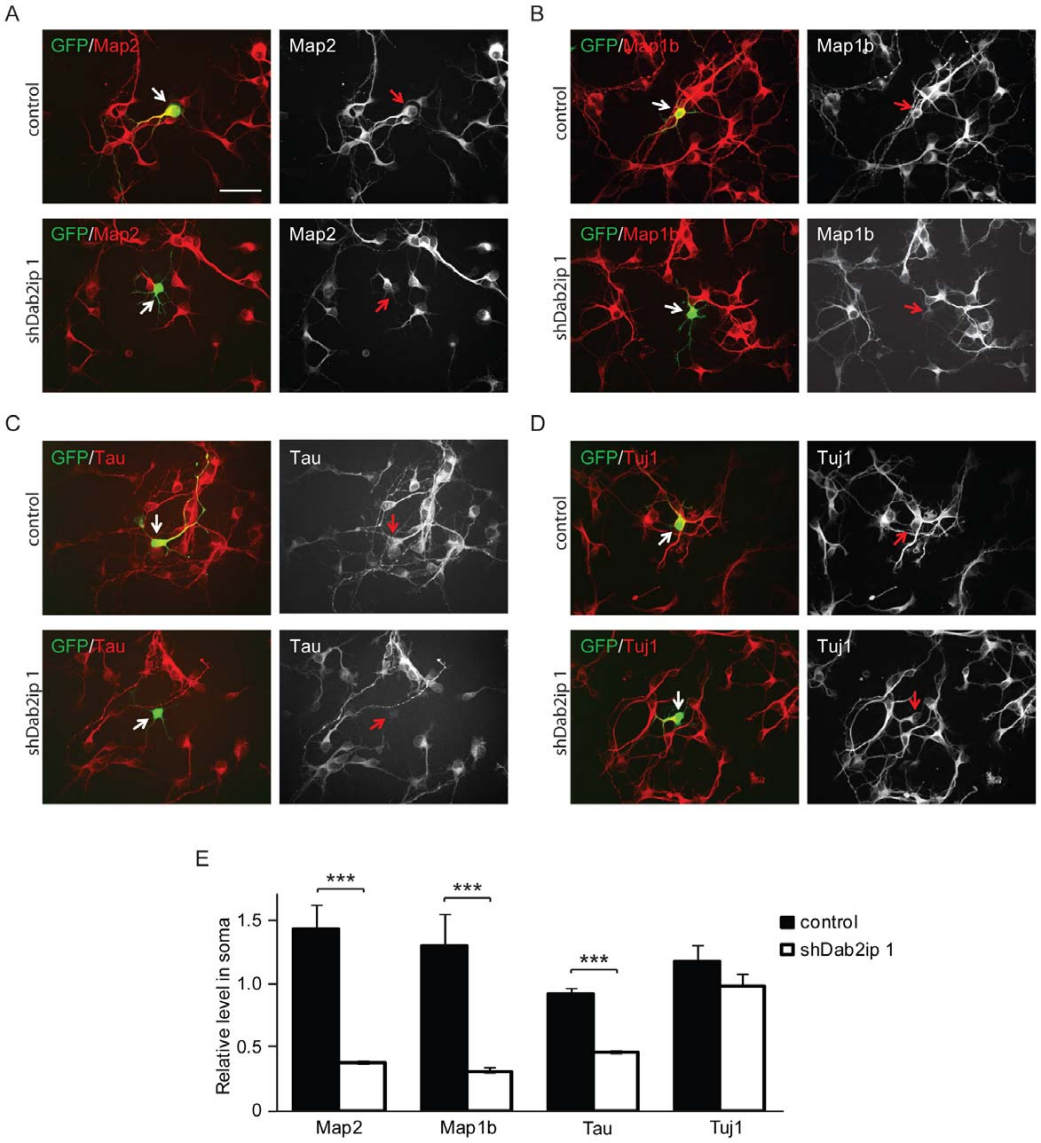
Dab2ip regulates the expression of microtubule-associated proteins. (A–D) Embryos were electroporated at E14.5 with control shRNA together with GFP and mCherry (double-labeled), or with shDab2ip 1 and GFP alone. Cortical neurons were isolated from transfected embryos at E16.5, cultured for 2 DIV, and subjected to immunofluorescence using Map2 (A), Map1b (B), Tau (C), and Tuj1 (D) specific antibodies, followed by Cy5-labeled secondary antibodies. Confocal images of fields containing neurons positive for GFP/mCherry or GFP alone were acquired. For simplicity, only the GFP and Cy5 channel are shown in the overlay images. Knock down neurons expressed undetectable or low levels of Map2, Map1b and Tau, but normal levels of Tuj1 compared to adjacent or control shRNA-transfected neurons. (E) Quantification of the data collected as in (A–D) from multiple experiments. The fluorescence signal of the soma of transfected neurons was measured relative to untransfected cells in the same field. Ten untransfected cells and 1 transfected cell were analyzed in each field from multiple fields. The data was pooled from >4 independent experiments. Scale bars: 50 µm ***, *p*<0.001.

Microtubules are structural components necessary for the acquisition of neuronal morphology, and microtubule-associated proteins (MAPs) are important for microtubule assembly, stabilization, and interaction with other cytoskeleton proteins [37], [38]. To investigate the molecular mechanisms underlying the effects of Dab2ip knock down on neurite development, we examined the expression of several MAPs by immunofluorescence in cultured neurons isolated from *in utero* electroporated embryos (IUE E14.5–E16.5). Immunofluorescence assays in cultured neurons revealed that Map2, a mature dendritic marker, was highly expressed in control (GFP+/mCherry+ double-labeled) neurons at 2 DIV, whereas it was expressed at very low levels in Dab2ip knock down (GFP+ only) neurons (Fig. 8A, E). The downregulation of Map2 in Dab2ip knock down neurons was apparent compared to either, adjacent untransfected neurons in the same culture wells, as well as compared to neurons transfected with control shRNA in parallel cultures. We also examined the expression of Map1b and Tau, two MAPs that *in vivo* are predominantly restricted to dendritic and axonal compartments, respectively. Our data indicate that the expression of Map1b and Tau is also strongly reduced in Dab2ip knock down neurons at 2 DIV (Fig. 8B, C, and E). The effect of Dab2ip on MAPs expression was rather specific, since levels of neuronal markers such as Tuj1 (Fig. 8D), Lis1, Dcx, and NeuN (data not shown) did not differ. The data suggests that Dab2ip regulates the expression of several MAPs during neuronal development and maturation.

## Discussion

In this study, we used *in utero* electroporation (IUE) to knock down or overexpress Dab2ip in the developing mouse neocortex and examined the role of this protein on neuronal migration *in vivo*. We also used cultured dissociated neurons *ex vivo* to further examine the effects of Dab2ip knock down on neurite extension. Our *in vivo* data suggest that normal levels of Dab2ip are necessary for the migration of late-born principal cortical neurons destined for upper cortical layers, which use glial-guided locomotion as their predominant modality of migration. Both reduced and increased Dab2ip levels *in vivo* resulted in defective migration, but the effects of the two manipulations on neuronal morphology were opposite: too little Dab2ip caused neurons to stall in the IZ with a multipolar morphology characteristic of neurons in this region, whereas too much Dab2ip caused neurons to become abnormally rounded and unable to acquire a multipolar morphology. We reasoned that Dab2ip plays a role in the transition from the multipolar to the bipolar stage of glial-dependent locomotion. This conclusion is further supported by *ex vivo* observations of the altered morphology of knock down neurons. For the conversion to a bipolar morphology in the upper intermediate zone, neurons likely require specific signaling events and cytoskeleton rearrangements. Indeed, proteins that affect microtubule dynamics have also been shown to be required in this step. For example, downregulation of Lis1 and doublecortin (Dcx) by IUE produced a phenotype quite similar to that observed here for Dab2ip [12]–[14]. However, in our hands re-expression of Dcx, albeit able to suppress the Dcx knockdown phenotype, did not rescue the Dab2ip knock down defect (unpublished observations). Furthermore, Dab2ip knock down did not affect Lis1 or Dcx expression (unpublished observations). Thus, it is likely that these proteins do not functionally interact with Dab2ip.

To control for potential non-targeting effects of our knock down constructs, we used a non-targeting shRNA control construct and 3 different Dab2ip shRNA constructs, which target different regions of the Dab2ip mRNA. All shDab2ip constructs resulted in similar migration defects, suggesting that the effects are specific. The phenotype resulting from Dab2ip knock down was dramatic and highly reproducible among several electroporated embryos from different litters. Thus, we conclude that Dab2ip expression is essential for proper cortical development. We also attempted to rescue the shDab1 migration defect by co-expressing mutagenized, non-degradable Dab2ip isoforms. However, these attempts were unsuccessful even when different plasmid amounts were used in the electroporation. We reasoned that the overexpression plasmids we used for the rescue either do not encode the proper isoform required for migration, or produce dominant-negative effects resulting from inappropriate levels or distribution of the exogenous Dab2ip protein. Indeed we found that overexpression of Dab2ip itself results in a migration defect, and that this phenotype is highly dependent on the presence of the PH domain, which promotes protein localization to the cell membrane. Since the intact GRD region of Dab2ip was also required for the migration phenotype in our overexpression experiments *in vivo*, the data suggest that Dab2ip normally functions at the membrane where it regulates the activity of GTP-binding proteins involved in cytoskeletal dynamics.

Previous studies demonstrated that early-born neurons destined to deep cortical layers migrate preferentially by somal translocation, whereas late-born neurons destined to upper cortical layers move into the cortical plate preferentially by glial guided locomotion, but also use somal translocation in the last phase of their migration. Our IUE experiments, coupled with layer-specific marker immunofluorescence, indicate that Dab2ip knock down preferentially affects the positioning of late-born neurons destined for upper layers. Because malpositioned knock down neurons born at E14.5 express the upper layer marker Satb2, and are surrounded by an apparently intact radial glia scaffold, we interpreted these data as an indication that Dab2ip affects their intrinsic ability to undergo glial-guided locomotion. However, neuronal migration may not be the only process that is affected by Dab2ip knock down, and the possibility that cell cycle exit and differentiation may also be affected cannot be presently excluded. Indeed, we noted that knock down neurons appear to leave the ventricular zone faster than control, consistent with premature differentiation (see Fig. S1). Further studies involving BrdU incorporation and additional markers of cell cycle progression and neuronal differentiation will be required to conclusively address this issue.

Radial migration in the developing neocortex is crucially regulated by Reelin, which is secreted by Cajal-Retzius cells in the marginal zone [7], [39], [40]. This protein functions by recruiting the adapter protein Dab1 in migrating principal neurons, which in turn engages downstream signaling molecules such as the PI3K [35], [41]–[44]. Dab1 protein levels are tightly regulated by Reelin activity through a mechanism that involves phosphorylation, ubiquitination and degradation [31]–[33], [45]. Even though Dab2ip interacts with Dab1 [20], [26], here we show that mutations in the Reelin pathway do not affect Dab2ip expression levels. Furthermore we showed that the NPxY Dab1-interacting motif is not necessary for Dab2ip to induce a migration defect *in vivo*. Together with the observation that Dab2ip knock down affects preferentially late-born neurons, whereas Dab1 functions in neurons that migrate by somal translocation [5], [6], these findings suggest that Dab2ip is not a positive mediator of Reelin activity. However, it would be premature at this point to conclude that Dab2ip is not involved at all in Reelin signaling. Dab2ip has been shown to possess a Ras GAP activity [20] and possibly Rap GAP activity depending on the cell type (S.H.K. and R.H. unpublished results). It is conceivable that Reelin may suppress the Rap1/Ras GAP activity of Dab2ip through Dab1 interactions, thus shifting the balance toward mechanisms that promote somal translocation at the expense of locomotion. Further biochemical and genetic studies will be necessary to investigate this possibility.

Microtubule associated proteins (MAPs) are important for microtubule assembly, stabilization, and interaction with other cytoskeleton proteins [37], [38]. The best-studied neuronal MAPs are Map2, Map1b and Tau. These proteins exist as several isoforms that are produced by alternative splicing, and their expression is developmentally regulated [46]–[49]. The function of Map2 and Tau are thought to be, at least in part, functionally redundant with Map1b during the early brain development. For example, knock down of either Map2 or Tau does not cause a significant defect in cortical development, including layer formation and neurite outgrowth [50], [51]. However, both Map2/Map1b and Map1b/Tau double knock out mice exhibit a neuronal migration defect and layer malformation as well as inhibited neurite growth. These deficits are more severe than the phenotype of single Map1b knock out mice [50]–[52]. Moreover, the combined down-regulation of these MAPs has been shown to affect neuronal polarity [53]. Our immunostaining results from cultured Dab2ip knock down neurons show that Dab2ip affects the expression levels of Map2, Map1b, and Tau, suggesting that the migration defect and morphological abnormalities seen in knock down neurons may be caused by the concomitant loss of these MAPs. Although the reduced expression of MAPs appeared to be transient, and these proteins gradually reappeared after 3 DIV (G.H.L. unpublished results), neurite development remained significantly altered in cultured Dab2ip knock down neurons, suggesting that even transient down-regulation of MAPs may have long-lasting consequences on neuronal maturation. The inhibition of MAPs expression also potentially explains the observed defects in glia-dependent locomotion, a process that is strongly dependent on the integrity of the leading edge. Further studies will be required to elucidate the exact molecular mechanism by which Dab2ip affects the expression of multiple MAPs.

In summary, we have demonstrated for the first time that Dab2ip plays a role in neuronal migration and neurite extension in the developing mammalian neocortex. These findings lay the foundation for further studies aimed at elucidating the molecular mechanisms of Dab2ip activity in the developing brain. The recent identification of Dab2ip in a set of genes that may contribute to the risk of autism and schizophrenia by affecting neurite outgrowth and guidance [54], [55] suggests that this protein could play surprisingly important roles in normal brain development as well as in neurodevelopmental brain disorders.

## Supporting Information

Figure S1 **Migration of neurons in the lateral neocortex following IUE.** Embryos were coelectroporated with GFP and control or shDab2ip 1 shRNA at E14.5. (A, C) Confocal images of GFP+ neurons in the developing necortex at E16.5 and at E17.5, respectively. (B, D) Plot profile of the images from the multiple samples. Scale bars: 200 µm, Control, *n* = 3; shDab2ip 1, *n* = 3.
(TIF)

Figure S2 **Expression of various Dab2ip constructs.** HEK293 T cells were transfected with no plasmid (−), a GFP expression plasmid control, or a series of mouse Dab2ip-L Myc constructs encoding wild type (WT) or the indicated mutant proteins. Western blot analysis of the cell lysates with Myc antibodies indicates that Dab2ip proteins are expressed at comparable levels. The appearance of non-specific bands at similar levels of intensity confirms equal protein loading.
(TIF)
