# Supplementary figures and images for: Dab2ip Regulates Neuronal Migration and Neurite Outgrowth in the Developing Neocortex
Source: PLoS One. 2012 Oct 4;7(10):e46592. doi: 10.1371/journal.pone.0046592 (PMC3464295; doi:10.1371/journal.pone.0046592)

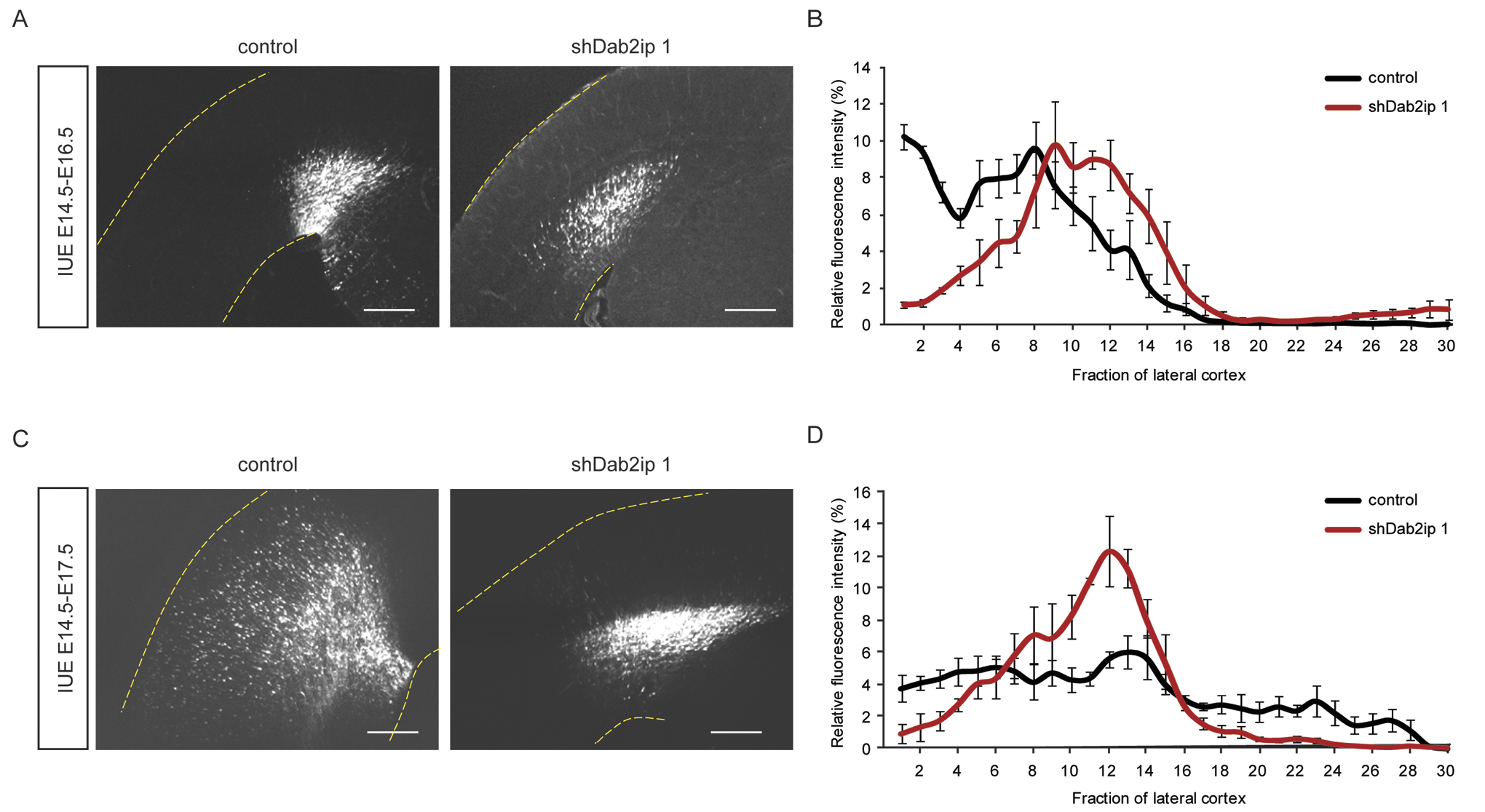

Supplement: Figure S1 — Migration of neurons in the lateral neocortex following IUE. Embryos were coelectroporated with GFP and control or shDab2ip 1 shRNA at E14.5. (A, C) Confocal images of GFP+ neurons in the developing necortex at E16.5 and at E17.5, respectively. (B, D) Plot profile of the images from the multiple samples. Scale bars: 200 µm, Control, n = 3; shDab2ip 1, n = 3. (TIF) [file pone.0046592.s001.tif]

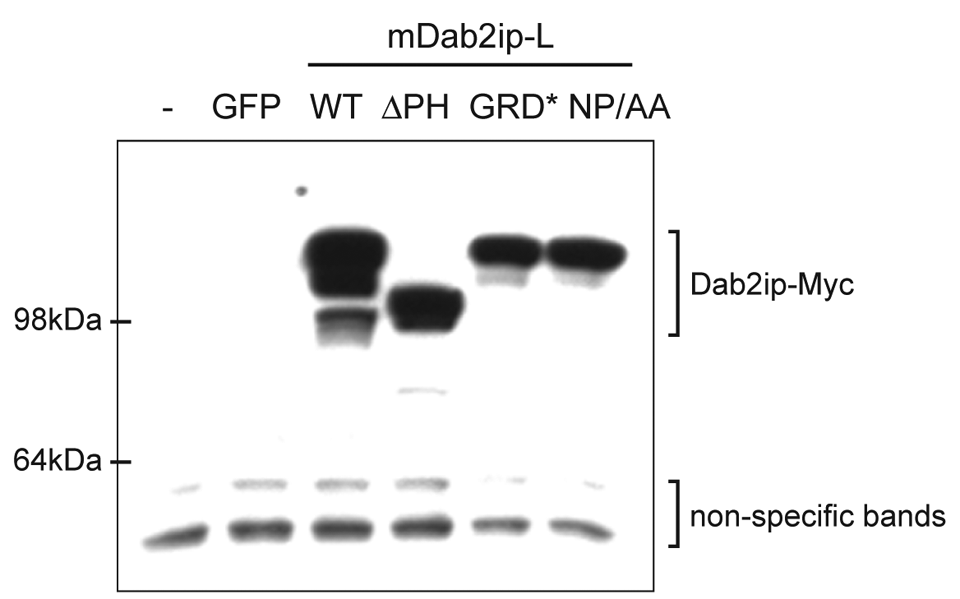

Supplement: Figure S2 — Expression of various Dab2ip constructs. HEK293 T cells were transfected with no plasmid (−), a GFP expression plasmid control, or a series of mouse Dab2ip-L Myc constructs encoding wild type (WT) or the indicated mutant proteins. Western blot analysis of the cell lysates with Myc antibodies indicates that Dab2ip proteins are expressed at comparable levels. The appearance of non-specific bands at similar levels of intensity confirms equal protein loading. (TIF) [file pone.0046592.s002.tif]
